# Marine Sponge-Derived Gukulenin A Sensitizes Ovarian Cancer Cells to PARP Inhibition via Ferroptosis Induction

**DOI:** 10.3390/md23040138

**Published:** 2025-03-22

**Authors:** Jin-Hyung Kim, Jung-Rae Rho, Jung-Hye Choi

**Affiliations:** 1 Department of Biomedical and Pharmaceutical Science, College of Pharmacy, Kyung Hee University, Seoul 02447, Republic of Korea; jenon@khu.ac.kr; 2 Department of Oceanography, Kunsan National University, Jeonbuk 54150, Republic of Korea; jrrho@kunsan.ac.kr; 3 Department of Integrated Drug Development and Natural Products, College of Pharmacy, Kyung Hee University, Seoul 02447, Republic of Korea

**Keywords:** Gukulenin A, olaparib, ovarian cancer, ferroptosis, lipid peroxidation

## Abstract

Resistance to PARP inhibitors (PARPi), such as olaparib (OLA), is a major challenge in ovarian cancer treatment. In this study, we investigated the combination effect of PARPi and gukulenin A (GUA), a bis-tropolone tetraterpenoid isolated from the marine sponge *Phorbas gukhulensis*. We found that GUA at a mildly cytotoxic dose synergistically enhanced OLA-induced cytotoxicity in human ovarian cancer cells. The combination treatment significantly increased reactive oxygen species (ROS) levels and lipid peroxidation, leading to ferroptotic rather than apoptotic cell death. Network pharmacology and gene ontology (GO) enrichment analyses revealed oxidative stress-related pathways as key mediators of this effect. Inhibition of NADPH oxidase (NOX) reversed combination-induced cell death, while ferrostatin-1 (FER-1), a ferroptosis inhibitor, significantly reduced lipid peroxidation and cytotoxicity. Additionally, GUA and OLA treatment suppressed ERK1/2 activation, and ERK overexpression attenuated the combination-induced cell death. Collectively, these findings suggest that marine-derived GUA enhances PARPi efficacy in ovarian cancer cells by inducing ferroptosis through oxidative stress and ERK pathway modulation.

## 1. Introduction

Ovarian cancer is the most lethal gynecologic malignancy, with high recurrence rates and limited treatment options [1,2]. The standard first-line therapy consists of surgical debulking followed by platinum- and paclitaxel-based chemotherapy [3]. However, despite initial responses, most patients experience relapse, leading to the urgent need for more effective treatment strategies. Recently, poly (ADP-ribose) polymerase inhibitors (PARPi) such as olaparib (OLA) have been approved for ovarian cancer patients with homologous recombination deficiency (HRD), including those with BRCA mutations [4]. While PARPi improved progression-free survival, the development of resistance remains a significant challenge [5]. Moreover, PARPi are less effective in patients without BRCA mutations, further limiting their clinical application [6]. Therefore, novel therapeutic strategies that can enhance PARPi sensitivity and overcome resistance mechanisms are highly desirable.

One promising approach to overcoming PARPi resistance is combination therapy with agents that can potentiate their efficacy [6]. Marine-derived bioactive compounds emerged as particularly promising due to their unique chemical structures and potent biological activities [7,8]. Notably, various marine-derived alkaloids, including guanidine alkaloids such as urupocidins A and C, demonstrated anticancer effects through mechanisms such as mitochondrial dysfunction and apoptosis induction, which complement the DNA damage-targeting effects of OLA [9]. Urupocidin C, in particular, exhibited strong synergy with OLA and has shown efficacy against drug-resistant cancer cells. Other marine-derived compounds, such as monanchoxymycalin C, crambescidins, and nortopsentins, exert anticancer activity through diverse mechanisms, including cell cycle modulation and lipid peroxidation, which may further enhance the therapeutic potential of PARPi-based therapies [10,11,12]. A well-established example is trabectedin (ET-743), a potent marine-derived compound isolated from the Caribbean tunicate *Ecteinascidia turbinata*, which has been shown to significantly enhance OLA efficacy in breast cancer models [13]. These findings underscore the potential of marine sponge-derived compounds in improving PARPi-based therapies.

Gukulenin A (GUA), a bis-tropolone tetraterpenoid isolated from the marine sponge *Phorbas gukhulensis*, has been shown to demonstrate a range of biological activities, including potential anticancer effects [14,15]. In a previous study, we also demonstrated that GUA effectively inhibits ovarian cancer cell proliferation and reduces tumor growth in vivo [16]. However, its potential role in overcoming PARPi resistance remains unexplored. Given the ability of marine-derived compounds to enhance OLA efficacy through diverse mechanisms, further investigation into the therapeutic potential of GUA in combination with OLA may provide valuable insights for overcoming PARPi resistance in ovarian cancer. In this study, we investigated the combinatorial effects of GUA and OLA in human ovarian cancer cells and the underlying molecular mechanisms.

## 2. Results

### 2.1. GUA-Mediated Enhancement of OLA Sensitivity in Human Ovarian Cancer Cells

**Figure 1:**
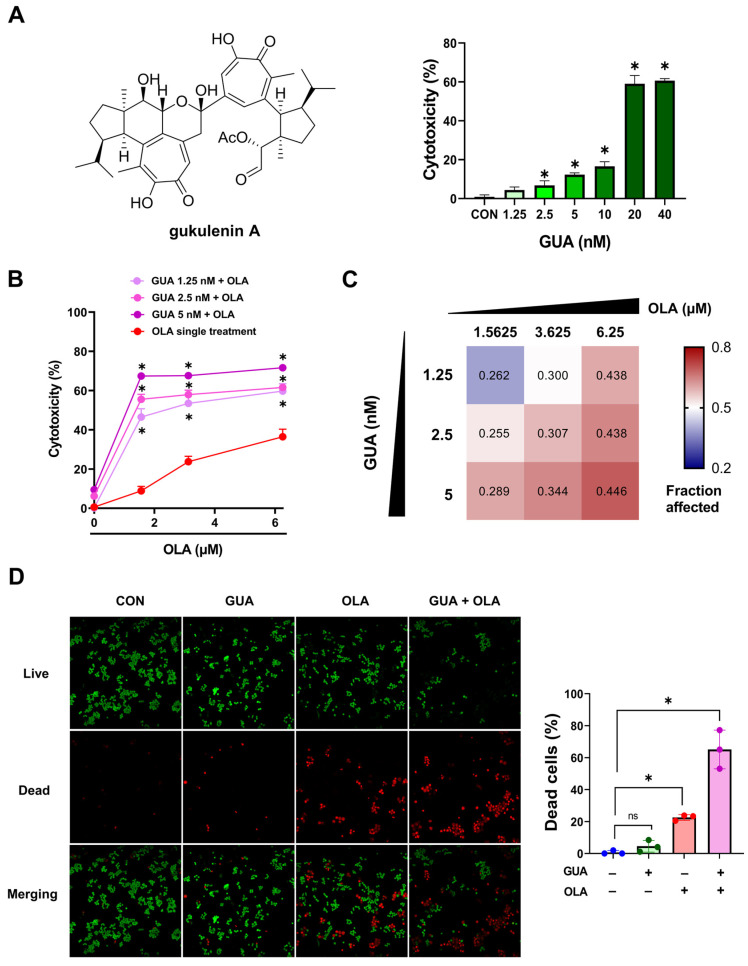
GUA enhances OLA sensitivity in ovarian cancer cells. (**A**) Chemical structure of GUA and its cytotoxicity profile in A2780 cells at varying concentrations, highlighting its subtoxic range. (**B**) Cell viability assessment following GUA-OLA combination treatment (GUA 5 nM and OLA 3 μM) for 48 h, demonstrating a significant dose-dependent reduction in viability compared to monotherapies. (**C**) CI values calculated via the Chou–Talalay method, confirming strong synergism (CI < 1) across tested concentrations. (**D**) LIVE/DEAD™ assay revealing a significant increase in dead cells under combination treatment (GUA 5 nM and OLA 3 μM) for 48 h. Green fluorescence represents live cells, and red fluorescence indicates dead cells. After a 24 h incubation, cells were treated with GUA (5 nM) and OLA (3 μM) for 48 h, imaged using a confocal fluorescence microscope, and analyzed with ImageJ software (version 1.54 g) Scale bar = 100 μm. CON, Control; GUA, Gukulenin A; OLA, Olaparib; and ns, not significant. The data represent three independent experiments. Statistical significance is denoted as * *p* < 0.05.

To evaluate the potential of GUA to enhance OLA sensitivity in ovarian cancer cells, we first determined a subtoxic dose of GUA (<20% cytotoxicity) for combination treatment (Figure 1A). Based on these results, we selected three GUA concentrations (1.25, 2.5, and 5 nM) and combined them with varying OLA doses (1.5625, 3.625, and 6.25 μM) (Figure 1B). The combination significantly enhanced OLA’s cytotoxicity, particularly at lower OLA concentrations (1.5625 to 3.625 μM), leading to a more than threefold increase in efficacy. To quantify the nature of these interactions, we calculated the combination index (CI) using the Chou–Talalay method. All tested combinations exhibited synergy, as indicated by CI values less than 1 (Figure 1C). To confirm these observations, we conducted a LIVE/DEAD™ assay, which demonstrated a significantly higher proportion of dead cells in the combination treatment compared to either agent alone (Figure 1D). Collectively, these results indicate that GUA enhances the cytotoxic efficacy of OLA in ovarian cancer cells.

### 2.2. Effects of GUA and OLA Combination Treatment on Cell Cycle Distribution and Apoptosis

**Figure 2:**
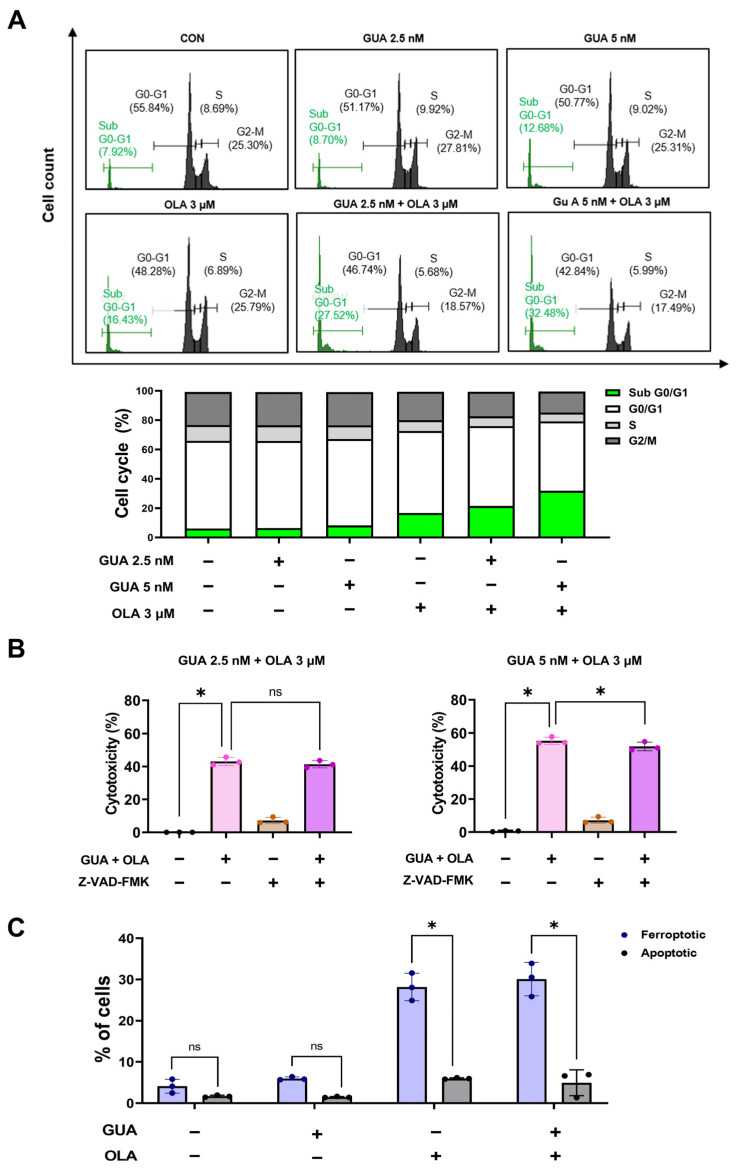
Effect of GUA-OLA combination on cell cycle dynamics and apoptosis. (**A**) Flow cytometry analysis of cell cycle phases illustrating a marked increase in sub-G0/G1 cell populations, indicative of cell death rather than classical cell cycle arrest. GUA (2.5 and 5 nM) and OLA (3 μM) for 48 h. (**B**) Cell viability after Z-VAD-FMK pretreatment (50 μM), followed by GUA (2.5 and 5 nM) and OLA (3 μM) combination treatment for 48 h, demonstrating that caspase inhibition does not significantly reverse cell death, suggesting a non-apoptotic mechanism. (**C**) Annexin V-FITC/PI staining confirming that apoptosis is not the primary mode of cell death following GUA-OLA co-treatment (GUA 5 nM and OLA 3 μM) for 48 h. ns, not significant. * *p* < 0.05.

To elucidate the mechanistic basis underlying the enhanced cytotoxicity observed with GUA-OLA combination treatment, we assessed cell cycle dynamics and apoptotic induction using flow cytometry. Treatment with GUA (2.5 and 5 nM) and OLA (3 μM) for 48 h resulted in a significant accumulation of cells in the sub-G0/G1 phase (Figure 2A), indicative of cell death rather than conventional cell cycle arrest. To delineate whether apoptosis is a primary driver of this cytotoxic response, we preincubated cells with the pan-caspase inhibitor Z-VAD-FMK. Notably, caspase inhibition did not substantially restore cell viability, suggesting that caspase-dependent apoptosis is not the predominant mechanism of cell death (Figure 2B). Furthermore, Annexin V-FITC/PI staining revealed that GUA did not significantly modulate the proportion of OLA-induced apoptotic cells (Figure 2C). These findings suggest that the observed cytotoxic effects of GUA-OLA co-treatment may be mediated through a non-apoptotic mechanism, necessitating further investigation to elucidate alternative pathways of cell death.

### 2.3. Network Pharmacology and GO Enrichment Analysis of GUA Targets in Ovarian Cancer

**Figure 3:**
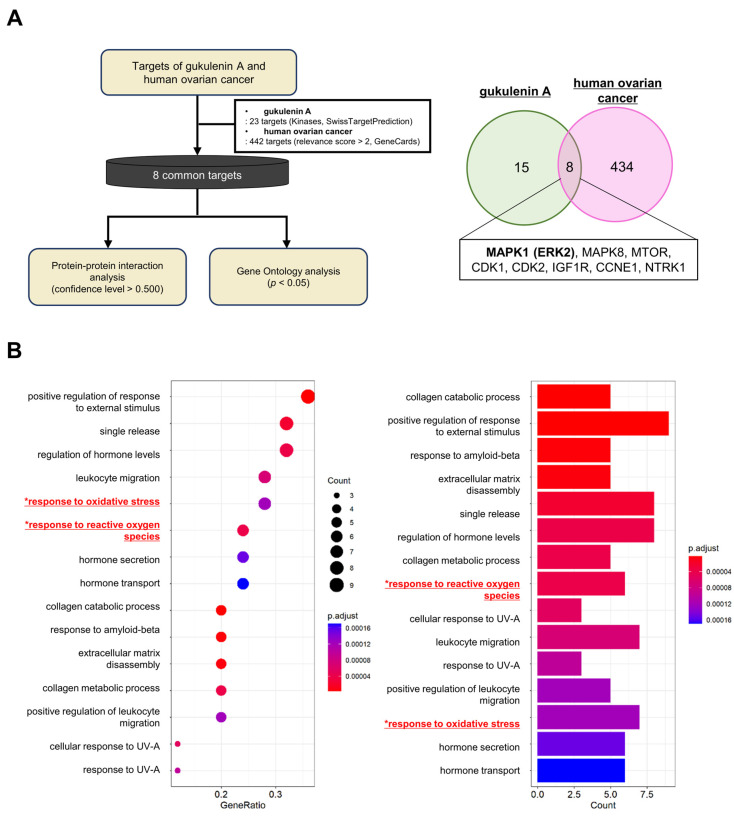
Network pharmacology and GO enrichment analysis of GUA targets in ovarian cancer cells. (**A**) Analysis workflow summarizing the integration of SwissTargetPrediction and GeneCards datasets to identify relevant molecular targets. Venn diagram depicting overlapping targets between GUA-associated proteins and ovarian cancer-related genes, highlighting eight key shared targets. (**B**) GO enrichment analysis emphasizing oxidative stress-related pathways, which were significantly enriched and implicated in GUA’s mechanism of action. The top 15 components (*p* < 0.05) are displayed, with the *Y*-axis showing the biological processes and the *X*-axis indicating gene ratios (dot plot) and the number of associated genes (bar plot).

To elucidate the molecular mechanisms underlying GUA’s activity, we performed a network pharmacology analysis (Figure 3A). Using SwissTargetPrediction, we identified 23 potential GUA targets, while GeneCards revealed 442 ovarian cancer-associated genes. Overlapping these datasets identified eight shared targets, suggesting their relevance in GUA-mediated anticancer effects.

Protein–protein interaction (PPI) network analysis of these targets highlighted interconnected roles in ovarian cancer pathogenesis (Figure S1). Gene ontology (GO) enrichment analysis identified 541 biological processes (BPs), with the top 15—including oxidative stress and ROS-related pathways—being significantly enriched (Figure 3B and Table S1). Given the strong association with oxidative stress, we next investigated ROS modulation in ovarian cancer cells.

### 2.4. Effects of GUA and OLA Combination Treatment on ROS Production and NOX

**Figure 4:**
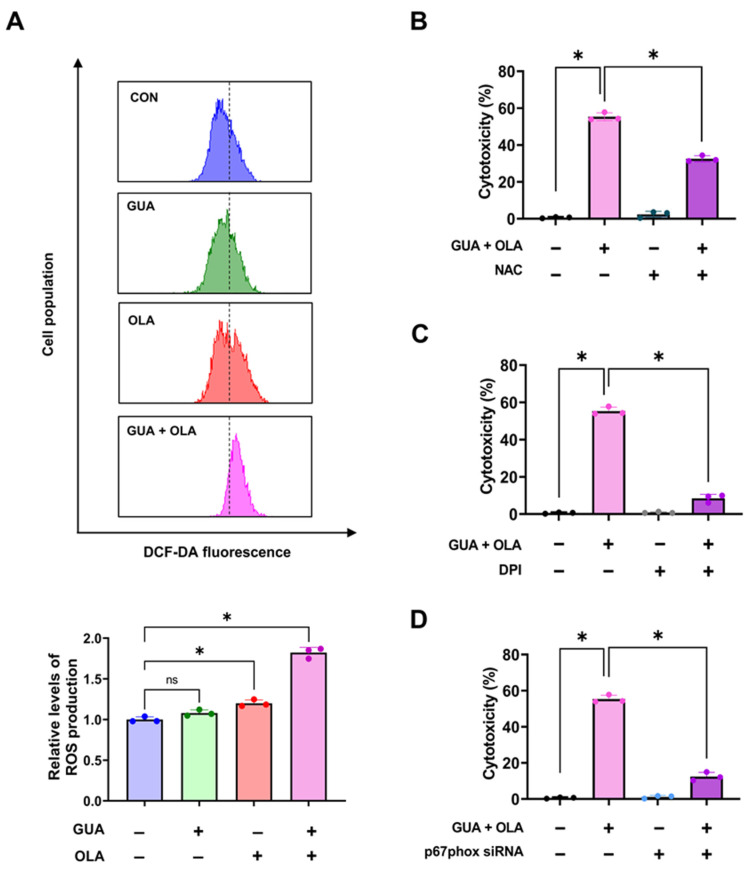
ROS production and the role of NOX in GUA-OLA-mediated cytotoxicity. (**A**) ROS levels assessed using DCFH-DA staining, indicating a substantial increase in oxidative stress upon combination treatment. GUA (5 nM) and OLA (3 μM) for 6 h. (**B**) NAC (5 mM) pretreatment for 2 h significantly rescues cell viability, confirming ROS-dependent cell death. (**C**,**D**) Effects of NOX inhibition (DPI, 0.1 μM) (**C**) and NOX subunit knockdown (p67phox siRNA 20 nM) (**D**), followed by 48 h treatment with GUA (5 nM) and OLA (3 μM), demonstrating a marked reduction in ROS levels and subsequent cell survival restoration, respectively. ns, not significant. * *p* < 0.05.

Given the enrichment of oxidative stress-related pathways, we hypothesized that GUA and OLA combination treatment enhances ROS production. 2′,7′-dichlorofluorescein diacetate (DCFH-DA) staining revealed significantly increased ROS levels upon combination treatment, whereas single-agent treatments had minimal effects (Figure 4A). Notably, pretreatment with the antioxidant *N*-acetylcysteine (NAC) significantly rescued cell viability (Figure 4B).

As NADPH oxidase (NOX) is a major source of ROS, we investigated its role in combination-induced cytotoxicity. Both NOX inhibition with diphenyleneiodonium chloride (DPI) and small interfering RNA (siRNA)-mediated knockdown of a NOX subunit p67phox significantly reduced cell death (Figure 4C,D), indicating that the combination treatment induces ROS-mediated cytotoxicity via NOX activation.

### 2.5. Involvement of Lipid Peroxidation in GUA and OLA Combination Treatment-Induced Ferroptosis

**Figure 5:**
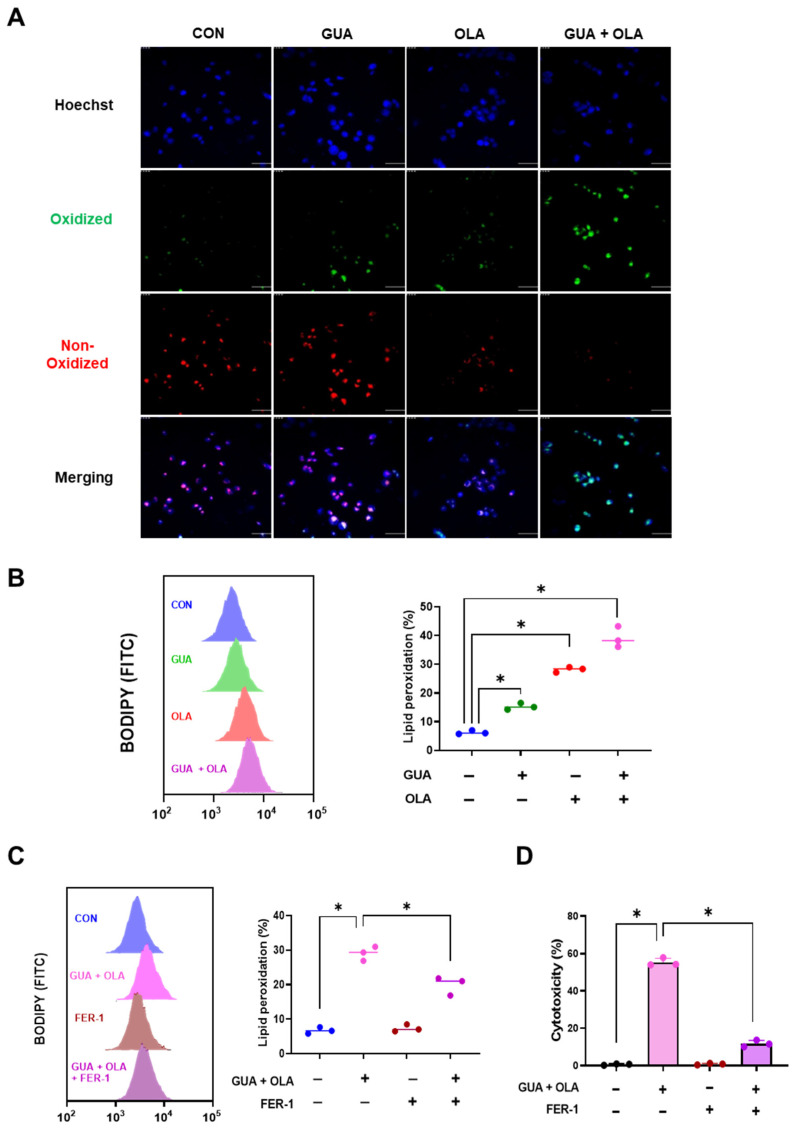
Lipid peroxidation as a key mechanism of ferroptosis induction in GUA-OLA treatment. (**A**) C11-BODIPY staining (2 μM) for 30 min, illustrating a significant increase in lipid peroxidation. GUA (5 nM) and OLA (3 μM) for 48 h. Scale bar = 50 μm. (**B**) Flow cytometric quantification of lipid peroxidation levels, revealing a time-dependent enhancement following treatment. (**C**) Ferroptosis inhibitor FER-1 (10 μM) for 2 h, followed by 48 h combination treatment with GUA (5 nM) and OLA (3 μM), mitigates lipid peroxidation, as evidenced by reduced C11-BODIPY oxidation. (**D**) Cell viability assay showing a substantial protective effect of FER-1, followed by 48 h combination treatment with GUA (5 nM) and OLA (3 μM), further supporting ferroptosis as the predominant cell death mechanism. * *p* < 0.05.

Ferroptosis, a form of regulated cell death driven by lipid peroxidation [17], has been implicated in OLA-induced cytotoxicity. To assess the involvement of ferroptosis, we measured lipid peroxidation using C11-BODIPY (581/591) staining. Combination treatment significantly increased C11-BODIPY oxidation, as indicated by the shift from red to green fluorescence (Figure 5A). Flow cytometric analysis confirmed a substantial increase in lipid peroxidation levels (Figure 5B). Moreover, time-course analysis demonstrated a progressive accumulation of lipid peroxidation, with a marked increase observed at 24 h post-treatment, suggesting a time-dependent induction of ferroptosis.

Furthermore, pretreatment with the ferroptosis inhibitor ferrostatin-1 (FER-1) significantly reduced lipid peroxidation (Figure 5C) and rescued cell viability (Figure 5D). These findings suggest that the GUA-OLA combination induces ferroptosis via lipid peroxidation.

### 2.6. Involvement of ERK Signaling in GUA and OLA Combination Treatment-Induced Ferroptosis

**Figure 6:**
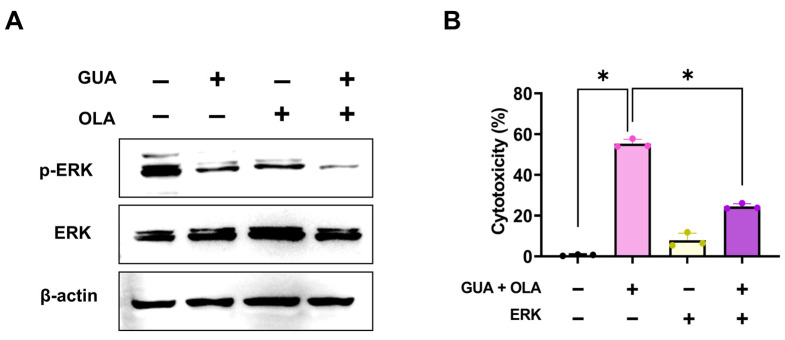
Involvement of ERK signaling in ferroptotic cell death. (**A**) Western blot analysis of p-ERK levels following GUA-OLA treatment, treated with GUA (5 nM) and OLA (3 μM) for 48 h, demonstrating significant suppression of ERK phosphorylation. (**B**) ERK overexpression partially rescues cell viability, indicating a mechanistic link between ERK inactivation and ferroptosis induction. Cells were transfected with ERK overexpression vector (10 nM) or control siRNA for 24 h, followed by 48 h combination treatment with GUA (5 nM) and OLA (3 μM). * *p* < 0.05.

GO analysis identified MAPK1 (ERK2) as a key oxidative stress-related gene. Western blot analysis revealed that combination treatment significantly suppressed ERK phosphorylation (Figure 6A). To assess the functional relevance of ERK signaling, we overexpressed ERK and observed partial rescue of cell viability (Figure 6B), supporting a role for ERK suppression in combination-induced ferroptosis. These results collectively indicate that the suppression of ERK activation contributes to ferroptosis in response to GUA-OLA combination treatment.

## 3. Discussion

Ferroptosis has been implicated in the anticancer effects of radiotherapy, immunotherapy, and specific chemotherapies [18]. Notably, chemo-resistant cancer cells are particularly vulnerable to ferroptosis due to enhanced DNA repair mechanisms or defects in apoptosis pathways [19]. Ferroptosis is triggered by the accumulation of peroxides derived from polyunsaturated fatty acid-containing phospholipids (PUFA-PLs), which overwhelm cellular defense systems [20]. This process is characterized by excessive ROS generation, lipid peroxidation, and iron accumulation [21]. The effective accumulation and insufficient clearance of lipid peroxides are essential for initiating ferroptosis, positioning it as a promising target for overcoming chemotherapy resistance in cancer cells [22]. A key finding of this study is that the GUA-OLA combination induces oxidative stress, leading to lipid peroxidation and ferroptosis. Our data demonstrate that this treatment overwhelms cellular antioxidant defenses, leading to the accumulation of lipid hydroperoxides, a hallmark of ferroptosis [23]. Specifically, the oxidation of membrane phospholipids results in a significant increase in lipid peroxidation, as indicated by elevated C11-BODIPY oxidation levels. The ferroptosis inhibitor FER-1 effectively mitigated lipid peroxidation and protected cells from ferroptotic cell death, reinforcing the role of lipid peroxidation in this process [24]. These findings highlight lipid peroxidation as the key mechanism underlying the cytotoxic effects of the GUA-OLA combination. Further investigation into the specific lipid peroxidation pathways targeted by GUA and OLA may provide deeper insights into their synergistic effects and potential therapeutic applications in ovarian cancer.

Beyond ferroptosis induction, our study revealed that the GUA-OLA combination primarily activates ROS-related pathways. This observation underscores the pivotal role of NOX-derived ROS in mediating the cytotoxicity of this combination. NOX enzymes contribute to tumor progression through various mechanisms, including oncogenic signaling activation, metabolic reprogramming, immune suppression, DNA damage, and metastasis [25,26,27,28,29]. Our findings demonstrate that the GUA-OLA combination significantly elevates NOX-derived ROS levels, which appears to be the primary driver of its cytotoxic effects rather than direct apoptosis induction. Since NOX-generated ROS can have contrasting roles in cancer, either facilitating tumor progression or causing oxidative stress-induced cell death, our findings suggest that the GUA-OLA combination effectively takes advantage of this susceptibility. The observed increase in NOX-derived ROS, coupled with the protective effects of NAC and the suppression of cytotoxicity by NOX inhibition, confirms that ROS generation is a critical mediator of the anticancer effects of this combination. This study emphasizes the significance of NOX-derived ROS in inducing cancer cell death and supports the potential of redox-based therapies to improve ovarian cancer treatment outcomes.

Furthermore, our study underscores the intricate relationship between ROS and ERK signaling in oxidative stress regulation. The GUA-OLA combination enhances NOX-derived ROS production while suppressing ERK phosphorylation (Figure 6A), a finding with significant downstream implications. ERK is a key regulator of cellular antioxidant defenses through activation of nuclear factor erythroid 2-related factor 2 (Nrf2), which modulates the expression of key antioxidant genes [30,31,32]. Reduced ERK activity may affect Nrf2 activation, potentially altering the cell’s ability to counteract oxidative stress [33]. Additionally, ERK inhibition may contribute to mitochondrial dysfunction and increased ROS generation, although this aspect requires further investigation [34]. The disruption of ERK signaling appears to drive lipid peroxidation, further promoting ferroptosis [23,35]. Our findings demonstrate that ERK overexpression rescues cells from the combination-induced cell death. This study suggests that NOX-derived ROS, through ERK suppression, may compromise cellular antioxidant defenses, thereby promoting lipid peroxidation and ferroptotic cell death.

In conclusion, this study explores the therapeutic potential of combining GUA, a marine-derived compound, with OLA, a PARPi, for ovarian cancer treatment. Our findings reveal that this combination triggers NOX-mediated ROS production, leading to ERK pathway inhibition, oxidative stress, and substantial lipid peroxidation, ultimately culminating in ferroptosis in ovarian cancer cells. The ability of GUA to enhance the efficacy of OLA highlights the potential of integrating natural products with targeted therapies. By elucidating the mechanisms by which oxidative stress and lipid peroxidation contribute to ovarian cancer cell death, this study reinforces ferroptosis as a promising therapeutic strategy and provides novel insights into redox-based approaches for ovarian cancer treatment.

## 4. Materials and Methods

### 4.1. Extraction and Purification of GUA

GUA, a compound of over 95% purity, was extracted from *Phorbas gukhulensis*, a marine sponge harvested near Gageodo, Republic of Korea, as detailed in our earlier study [16]. The extraction process involved sequential partitioning of the methanol extract between dichloromethane and water, followed by 15% aqueous methanol and n-hexane. The resulting polar fraction underwent reverse-phase flash column chromatography, yielding 7 subfractions. The bioactive subfraction was further purified via reverse-phase HPLC, isolating GUA. The purity of GUA was determined to be over 95%, as confirmed by the proton NMR spectrum (Figure S2), which was used in place of a chromatogram. Spectroscopic and chemical analyses identified GUA as a bis-tropolone tetraterpenoid.

### 4.2. Materials

The MTT reagent was purchased from Thermo Fisher Scientific (Waltham, MA, USA). Small interfering RNA targeting p67phox was obtained from Bioneer Technology (Seoul, Republic of Korea). OLA was purchased from Selleckchem (Houston, TX, USA). FER-1 and DPI were supplied by MedChemExpress (Monmouth Junction, NJ, USA), while NAC and C11-BODIPY (581/591) were acquired from Sigma-Aldrich (St. Louis, MO, USA). Broad caspase inhibitor Z-VAD-FMK was obtained from Calbiochem (Bad Soden, Germany). Roswell Park Memorial Institute (RPMI) 1640, penicillin, and streptomycin were sourced from WELGENE Inc. (Gyeongsan, Republic of Korea). Fetal bovine serum (FBS) was procured from Hyclone (Logan, UT, USA). Antibodies including phospho ERK, ERK, and β-actin antibody, along with DCFH-DA, were acquired from Cell Signaling Technology (Danver, MA, USA) and Santa Cruz Biotechnology (Dallas, TX, USA).

### 4.3. Cell Culture and Cell Viability/Cytoxicity Assay

A2780 human ovarian cancer cell line, obtained from the American Type Culture Collection (ATCC; Manassas, VA, USA), was maintained in RPMI 1640 medium enriched with 5–10% FBS and antibiotics (100 U/mL penicillin and 100 μg/mL streptomycin). Cells were cultured at 37 °C in a 5% CO_2_ humidified incubator. For viability assays, A2780 cells were plated in 96-well plates at 1.0 × 10^5^ cells per well. Following overnight attachment, cells were treated with GUA or OLA for 48 h. Cell viability was assessed using the MTT assay. Briefly, MTT solution (1 mg/mL) was added to each well and incubated for 4 h. The resulting formazan crystals were solubilized in DMSO, and absorbance was measured at 540 nm using a SpectraMax microplate spectrophotometer (Molecular Devices, Sunnyvale, CA, USA). To measure cytotoxicity, the LIVE/DEAD™ Assay Kit (Life Technologies Corporation, Johannesburg, South Africa) was used according to the manufacturer’s instructions to qualitatively visualize the distribution of viable and non-viable A2780 ovarian cancer cells after treatment with GUA and OLA. Briefly, the cells were stained with 1 mL of 1× PBS containing 1 µL of calcein and 2 µL of ethidium homodimer-1 (EthD-1) for 30 min. The stained cells were then visualized using Alexa Fluor 488 and EtBr filters under a confocal fluorescence microscope.

### 4.4. Annexin-V-FITC and PI Staining Assay

Apoptosis and cell cycle analysis were performed using flow cytometry. For apoptosis detection, cells were stained with the ApoScan^TM^ Annexin-V-FITC Apoptosis Detection Kit (BioBud, Suwon, Republic of Korea), following the manufacturer’s protocol. Briefly, treated cells were washed with PBS, incubated with FITC-conjugated Annexin-V for 15 min in the dark, and then stained with PI. For cell cycle analysis, A2780 ovarian cancer cells were collected, washed, and stained with PI. Both apoptosis and cell cycle distribution were analyzed using a Beckman Coulter^®^ flow cytometer (Brea, CA, USA), with PI fluorescence detected via the FL-2 or FL-3 channel to quantify DNA content and determine cell cycle phases.

### 4.5. Transfection

For transfection, cells were cultured in 6-well plates at 0.8 × 10^5^ cells/wells and incubated at 37 °C. After 24 h, each transfection mixture was prepared by mixing the 10 nM (p67phox siRNA and control siRNA), 5 μL INTERFERin reagent (Illkirch, France) in serum-free Opti-modified eagle’s medium (Opti-MEM).

### 4.6. Network Pharmacology and GO Enrichment Analysis

The 23 putative targets of GUA were collected from the SwissTargetPrediction (https://www.swisstargetprediction.ch, accessed on 23 May 2024). Human ovarian cancer-related genes with relevance scores greater than over 2 were extracted from the GeneCards database (https://www.genecards.org, accessed on 21 November 2024). PPI analysis was performed on the common genes of the putative targets of GUA and human ovarian cancer-related genes using the STRING database (https://www.string-db.org/, accessed on 23 November 2024). The edges of the PPI network were constructed based on a confidence level greater than 0.5. GO enrichment analysis was performed on the BPs using the clusterProfiler package of the R software (version 4.3.1), and the results are visualized in a dot and bar plot.

### 4.7. Detection of Intracellular ROS

Intracellular ROS levels were measured using a fluorescent probe DCFH-DA. Treated cells were stained with DCFH-DA (100 μM) for 30 min in darkness, washed with PBS, and analyzed using a CytoFLEX flow cytometer (Beckman Coulter Life Science, Miami, FL, USA).

### 4.8. Lipid Peroxidation Assay

Lipid peroxidation was assessed using both flow cytometry and confocal laser scanning microscopy. For flow cytometric analysis, cells were seeded in 6-well plates and allowed to adhere overnight before treatment with the specified agents. Subsequently, cells were incubated with 10 μM C11-BODIPY (581/591) (Thermo Fisher, D3861) for 30 min, washed with PBS, trypsinized, and resuspended in PBS prior to flow cytometry analysis. For confocal imaging, cells were cultured in a 4-chamber glass bottom dish (SPL Life Sciences, Pocheon, Republic of Korea) and treated as previously described. Cells were then incubated with 5 μM C11-BODIPY (581/591) for 30 min, washed with PBS, and imaged using a K1-Fluo confocal fluorescence microscope (Nanoscope Systems Inc., Daejeon, Republic of Korea).

### 4.9. Western Blot Analysis

Cell pellets were washed, lysed, and subjected to SDS-PAGE. Proteins were transferred to PVDF membranes, incubated with antibodies, and detected via ECL chemiluminescence. Band intensity was analyzed using ImageJ (version 1.54 g).

### 4.10. Statistical Analysis

Data are shown as mean ± SD. Statistical tests were carried out using GraphPad Prism software 8.0 (GraphPad Software, Inc., La Jolla, CA, USA). Depending on the analysis type, one-way ANOVA, two-way ANOVA, or Student’s *t*-test was applied, with significance defined as *p* < 0.05.

## Data Availability

The data underlying the findings of this study can be obtained from the corresponding author upon reasonable request.

## Supplementary Materials

The following supporting information can be downloaded at: https://www.mdpi.com/article/10.3390/md23040138/s1, Table S1: GO functional enrichment analysis of GUA with the top 15 BPs including cellular responses to oxidative and ROS stresses. Figure S1: PPI network of the 8 shared targets, with nodes representing proteins and edges denoting interactions between them. Figure S2: ^1^H-NMR spectrum of GUA.

## Abbreviations

ATCC: American Type Culture Collection; BP: Biological Process; BRCA: Breast Cancer gene; CI: Combination Index; DCFH-DA: 2′,7′-dichlorofluorescein diacetate; DPI: Diphenyleneiodonium chloride; ERK: Extracellular signal-Regulated Kinase; EthD-1: Ethidium homodimer-1; FBS: Fetal Bovine Serum; FER-1: Ferrostatin-1; FITC: Fluorescein Isothiocyanate; GO: Gene Ontology; GUA: Gukulenin A; HPLC: High-Performance Liquid Chromatography; HRD: Homologous Recombination Deficiency; MAPK1: Mitogen-Activated Protein Kinase 1 (ERK2); MTT: 3-(4,5-Dimethylthiazol-2-Yl)-2,5-Diphenyltetrazolium Bromide; NAC: *N*-acetylcysteine; NOX: NADPH Oxidase; Nrf2: Nuclear factor erythroid 2-related factor 2; OLA: Olaparib; Opti-MEM: Opti-modified eagle’s medium; PARP: Poly (ADP-ribose) polymerase; PARPi: PARP inhibitors; PBS: Phosphate Buffered Saline; PI: Propidium Iodide; PPI: Protein–Protein interaction; PUFA-PLs: Polyunsaturated Fatty Acid-containing Phospholipids; PVDF: Polyvinylidene Fluoride; ROS: Reactive Oxygen Species; RPMI 1640: Roswell Park Memorial Institute 1640 medium; SDS-PAGE: Sodium Dodecyl Sulfate–Polyacrylamide Gel Electrophoresis; and siRNA: Small interfering RNA
