# Supplementary material for: Marine Sponge-Derived Gukulenin A Sensitizes Ovarian Cancer Cells to PARP Inhibition via Ferroptosis Induction
Source: Mar Drugs. 2025 Mar 22;23(4):138. doi: 10.3390/md23040138 (PMC12028354; doi:10.3390/md23040138)
Supplement: Supplementary file 1 [file marinedrugs-23-00138-s001.zip › Supplementary Figures.pdf]

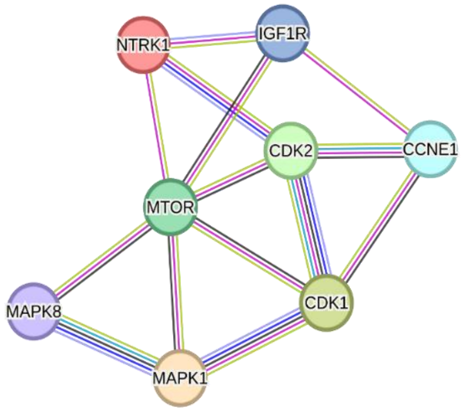

Supplementary Figure S1. Protein-Protein Interaction (PPI) Network of GUA Targets in Human Ovarian Cancer

The protein-protein interaction (PPI) network of the 8 shared targets was constructed using the STRING database, with nodes representing proteins and edges denoting interactions between them.

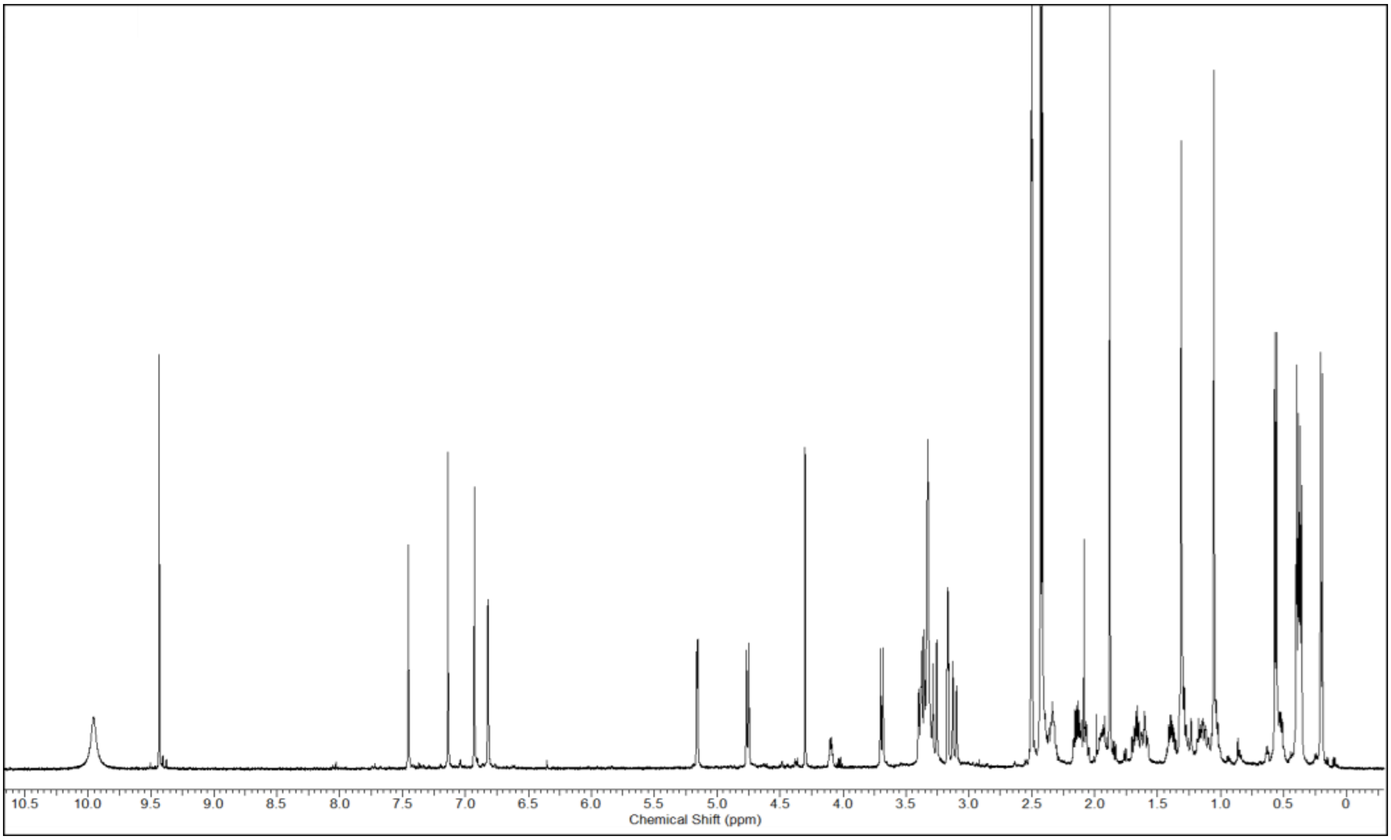

Supplementary Figure S2. 1H-NMR Spectrum of Gukulenin A (GUA)
